# Diagnostics and Training of Affordance Perception in Healthy Young Adults—Implications for Post-Stroke Neurorehabilitation

**DOI:** 10.3389/fnhum.2015.00674

**Published:** 2016-01-06

**Authors:** Jennifer Randerath, Scott H. Frey

**Affiliations:** ^1^Department of Psychology, University of KonstanzKonstanz, Germany; ^2^Lurija Institute for Rehabilitation Science and Health Research, Kliniken SchmiederAllensbach, Germany; ^3^Department of Psychological Sciences, University of MissouriColumbia, MO, USA; ^4^Program in Occupational Therapy, Department of Neurology and Department of Psychological and Brain Sciences, Washington University School of MedicineSt. Louis, MO, USA

**Keywords:** affordance perception, perception-action, training, detection theory, transfer

## Abstract

Affordance perception is critical to adaptive behavior. It comprises the ability to evaluate whether the environment and the actor's capabilities enable particular actions. It remains unclear how brain damage and its behavioral sequela impact this ability. Two affordance based judgment tasks were applied in healthy young adults that were adapted for prospective diagnostic purposes in patients. In addition to the commonly analyzed error-rate we included response times and accuracy measures based on a detection theory approach. Moreover, a manipulation was added intended to determine the effectiveness of feedback-based learning. We further applied control tasks that consider whether errors in affordance perception can be explained by errors in perception. Participants responded yes or no to decide prospectively if a given setting would afford a particular action. In study1, 27 participants judged whether their hand would fit through a given aperture (adapted from Ishak et al., 2008). In study2, 19 participants judged whether objects are reachable [adapted from Gabbard et al. (2005)]. For both studies two sessions were administered. In the first session all participants solved the judgment-task without executing the action. In the second session (feedback manipulation), half of the participants were allowed to first judge and then perform the task for each trial (reach forward and touch the object, or fitting the hand into the aperture). Judgments were slowest and errors most frequent for openings or distances close to the individual's actual physical limits. With more extreme settings accuracy increased and responses became faster. Importantly, we found an advantageous effect of feedback on performance in both tasks suggesting that affordance perception is rapidly trainable. Further, the aperture task demonstrated that feedback experienced with one hand can transfer to the other. This may have important implications for rehabilitation.

## Introduction

Affordance perception comprises the perception of action opportunities, including processing the properties of the environment as well as one's own capabilities (Gibson, 1979). The theory of affordances points out the close relationship between perception and action. When navigating through our environment and interacting with tools and objects it is necessary to prospectively adapt our movement plan based on what we perceive. Appropriate affordance perception supports us in determining what actions we can and will execute. On the other hand it also helps determining what actions to avoid, when the environment or our bodily capabilities do not provide the appropriate conditions. Despite the tendencies to overestimate or underestimate abilities for certain tasks, healthy young adults are perfectly able to perform appropriate decisions such as when reaching for objects (Carello et al., 1989; Gabbard et al., 2005, 2006), passing between obstacles (Wagman and Malek, 2008; Higuchi et al., 2009) or fitting the hand into an aperture (Ishak et al., 2008). Few studies investigated the effects of training and exposure to actions on affordance based judgments. The results thus far are promising in that learning and improvements in these tasks have been demonstrated for healthy young adults. After training and exposure they show quick adaptation to new constraints (Mark and Vogele, 1987; Mark et al., 1990; Weast et al., 2011).

The function affordance perception is critical to adaptive behavior. Major misjudgments of action opportunities could lead to precarious situations, including such mishaps as limb injuries and falls. While slight misjudgments in healthy young adults may not breach safety boundaries, this may be different in patients with brain damage. When seated, healthy subjects for example typically overestimate what they can reach (Carello et al., 1989; Mark et al., 1997; Gabbard et al., 2011). Yet, they seem to adequately adapt their estimation criterion within their safety boundaries. For reachability judgments Gabbard et al. (2007) for example found less overestimation to be apparent in a standing vs. seated condition. The authors attribute this more conservative estimation in the standing position to greater perceived postural demands.

It is feasible that brain damage and resulting lost functions may affect adequate affordance perception, and in patients the tendency to be out in their estimation could be magnified dramatically. There is some evidence that the likelihood for falls may increase. For example, errors in perceiving postural limits by estimating the maximum reach of the non-affected side of hemiplegic patients correlates with high risk for falling (Takatori et al., 2009). However, neither the incidence for potentially undiagnosed deficient affordance perception after stroke nor the potential underlying mechanisms are thus far enlightened. It is therefore important to provide tools for diagnostics and training in affordance perception for patients with brain damage.

Notably, the picture of impaired affordance perception may be complex, since brain damage could affect this ability on diverse levels. First, brain damage can change bodily capabilities, for example by causing hemiplegia. These new body constraints have to be taken into account when planning and executing actions. Interestingly, some (Johnson, 2000; Johnson et al., 2002), but not all (Buxbaum et al., 2005) stroke patients retain the ability to plan movements that have become impossible due to hemiplegia with remarkable accuracy. This has a potential downside, as it could lead to attempting now impossible actions and precipitate costly errors including failed actions, unstable postures and falls. Second, impaired cognitive functions may correlate with the ability to adequately perceive affordances. Left brain damage due to stroke can lead to problems in action planning (Rushworth et al., 1998; Buxbaum et al., 2005; Sunderland et al., 2011), which typically is attributed to malfunctions of a left lateralized praxis network. The associated cognitive motor disorder summarizing resulting problems like selecting or producing inappropriate actions is called limb apraxia (Goldenberg, 2013). One underlying mechanism may be the impaired integration of information into an action plan, that includes processed information about environmental properties and own body parts (Frey, 2007; Randerath et al., 2009, 2011). These are essential aspects of the concept of affordance perception, thus limb apraxia may correlate with disturbances therein. Further, right brain damage may lead to visuo-spatial neglect and impair the perception of spatial properties in the contralesional hemispace (Karnath et al., 2011). Interestingly, several studies demonstrated that actual reaching or grasping even in the contralesional field is similar to that in controls or patients without neglect (Himmelbach and Karnath, 2003; Mcintosh et al., 2004; Harvey and Rossit, 2012). Yet, while these studies indicate that neglect patients perform relatively better in action tasks, their severe visuospatial impairments may affect prospective judgments about action opportunities. Both, new constraints as well as cognitive disabilities may affect affordance perception in stroke patients.

Attempts to study possible disruptive effects of impairments after stroke on prospectively judging action opportunities are scarce. We here present a paradigm applied in healthy young adults, which measures the ability to judge action opportunities. With the future goal to evaluate preserved or impaired affordance perception in the stroke-patient population, our approach takes known challenges such as aphasia, neglect and hemiparesis into account by using simple instructions, limited number of trials (doable within 30 min) and factoring in difficulties with attention to the contralesional hemi-space as well as the use of only one hand. Moreover, the paradigm includes the possibility of training and thus potentially improving behavior.

In addition to typical accuracy percentages we used a detection theory approach to analyze our data. When affordance based judgments are required, subjects' decision making may be influenced by a number of different factors including their perceptual sensitivity and their response biases. Participants may decide whether an action is possible by comparing observations with a criterion. We therefore calculated subjects' discriminability, response bias, and diagnostic accuracy with the help of detection theory (Green and Swets, 1966; Fox, 2004; Macmillan and Creelman, 2004). Further, we tested for positive effects of task exposure on affordance based judgments, and we explored whether feedback presented to one side of the body may be transferred to the other side.

In two studies we evaluated response time (RT) and accuracy measures for separate tasks (study1: aperture-task; study2: reach-task) without and with feedback. Each study required healthy young adults to make judgments about body-object relationships. For both studies two sessions were administered. In the first session participants judged (yes or no) whether for a given setting a particular action is possible (study1, aperture-task: fitting the hand into the aperture; study2, reach-task: touching the object). In the second session, half of the participants were allowed to first judge and then actually perform the task for each trial (Experimental-group), and the other half (Control-group) once more merely judged whether the given setting allows the particular action. In study1 the task was to judge whether the hands can fit through an aperture. In study2 we looked at how well seated people determine if an object is within their reach while bending forward is allowed. Hence, the latter task introduces more degrees of freedom.

Participants were confronted with a fixed set of increments based on the individual's capabilities (maximum reach or smallest possible aperture to fit in) measured at the beginning of each session^1^. With this approach it has been shown that for certain increments reachability judgments vary within individuals. Healthy young adults have no problems judging items that are located further away from their actual reach limit, but for items positioned very close to the actual reach limit error-rates are close to chance (Gabbard et al., 2007). In the current study, we predicted that judgments are slower and less accurate when decisions have to be made for settings that are closest to the actual physical limits compared to more extreme settings,—independent from the used paradigm (opening width or object distance). Significant increases in accuracy due to feedback were expected for the Experimental-group only in the second compared to the first session.

We further expected affordance perception to engage a complex network of components involved in motor cognition. Perception of environmental properties (such as size or distance) is one of these facets and may correlate with the ability to judge action opportunities. In order to determine the relationship we added a size adjustment-task to the aperture paradigm and a depth perception task to the reachability paradigm.

## Study 1: aperture task

### Method

#### Participants

Twenty seven individuals (14 female; mean age = 21 ± 3.7 years) from the University of Oregon participated in the two-session study. All participants were right-handed according to the Edinburgh Handedness Inventory (Oldfield, 1971), had normal or corrected-to-normal vision (at least 30 ft/9 m), and were naïve to the specific goals of the study. Participants provided informed consent in accordance with the local IRB and the Declaration of Helsinki. The study took approximately 2 h to complete (45–60 min per session). Participants received financial or study credit compensation. Next to the experimental tasks (approximately 30 min) they completed the consent-form, a handedness questionnaire, a vision test and received study-debriefing. Half (*N* = 14) of the group were assigned to an Experimental-group receiving feedback in the second session, the other half served as Control-group. Participants were randomly assigned.

#### Materials and procedures

##### Material

**Figure 1:**
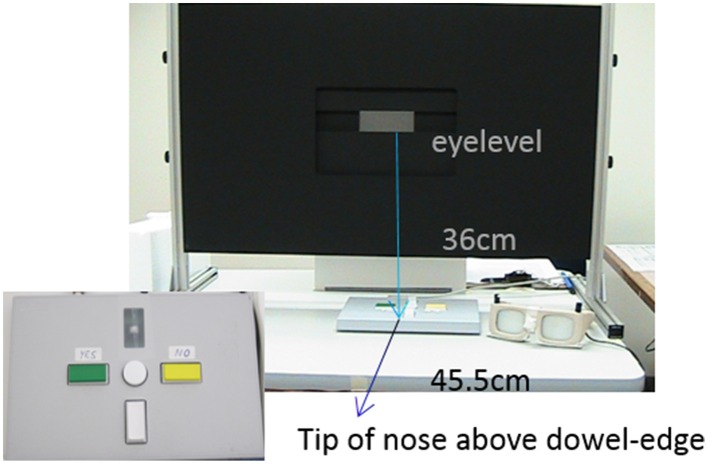
**Setting for the aperture task**. Participants were seated centrally in front of the apparatus, with the eyes being on the level of the opening and their tip of the nose being positioned vertically in line with the edge of a dowel fastened below the table (about 45.5 cm from the apparatus). Manual adjustments of the aperture were possible using a belt mechanism in the back of the apparatus; dimension specifications allowed for measuring millimeter increments. Participants wore plato-goggles (here: opaque) throughout the experiment to control for visual feedback and to allow for RT-measurements. Goggles were opened for the start of each trial. The rightwards shifted positioning of the response box reflects an affordance judgment block for active right hand judgments.

The aperture apparatus was custom made for this project and mounted on a height adjustable table (Figure 1). The width and height of a centrally placed rectangular opening was manipulated manually by the experimenter with knobs on the back of the device. The participants' hands (left = LH, right = RH) served as stimuli for two tasks—a size perception and an affordance based judgment task (Aperture task). Participants wore plato-goggles throughout the experiment to control for visual feedback and to allow for response time measurements (Translucent Technologies Inc.).

##### Measurements

Each session started out with measuring the width of the two hands in the aperture by closing the opening tightly around the flat hands with fingers closely spaced. For the measuring procedures the goggles were turned opaque to avoid visual feedback. The hands were measured at the widest part which was defined as the horizontal distance from the outer side of the pinky to the outer side of the thumb, the measurement was taken at the transition of the proximal phalanges and metacarpal bones. During the sessions the vertical opening size was always set to the thickness of the individual's hand to be judged upon.

##### Affordance based judgments

In the so called Aperture task participants had to determine whether they would be able to fit the hand through a given aperture. The horizontal width of the opening was varied using fixed negative and positive increments according to each hand's size (opening: −1.6, −0.8, −0.4, −0.2, ±0, +0.2, +0.4, +0.8, and +1.6 cm). Participants solved 36 (4 × 9 openings) plus 4 filler trials per hand, resulting in a total of 80 trials. The 0-trials reflected the measurement, i.e., the smallest possible size of the opening when the hand could fit into the aperture. To avoid an imbalance toward more frequent yes-trials, we added filler trials for which the correct answer would be “No” (smaller than −1.6 cm).

It was blocked whether judgments were made for the left or right hand (group A/B: LRLR or RLRL). Blocked presentation was used in anticipation of applying this paradigm in stroke patients with potential hemiparesis, who are not able to change the hand's position frequently. Furthermore, stroke patients may be restricted to use their unaffected hand for indicating their judgments with a button press response. Thus, in the current study our healthy participants were assigned to two groups: Half of the group indicated their responses via button press with their left hand, the other half with their right hand. That way we could analyze whether hand-dominance may play a role when indicating the response. Participants always started with a block of judgments for the assigned button pressing hand, then they judged the other hand (while indicating the response with the assigned hand). Here we name the hand that always indicates button responses the *active hand*. The other hand is called the *passive hand*. Stimuli were positioned onto a mark. If the active hand served as stimulus, the response-box was set on top of the stimulus-mark,—if the passive hand was judged the response-box with the active hand was moved 8 cm toward the outer edge of the apparatus to allow for the passive hand to be positioned on the stimulus-mark. The stimulus-mark was slightly shifted from the aperture's midline (3.5 cm) to avoid direct alignment strategies. In anticipation of applying this paradigm in stroke patients with potential neglect, the shift was yoked with the group either toward left hemispace (group A, left hand active) or right hemispace (group B, right hand active). Before each block participants were reminded about what hand they next had to base their judgments on. Each block started with 2 demonstration trials, presenting an extreme small or wide aperture. Summarized, participants solved 4 blocks judging whether one specified hand could fit into a given opening, two blocks of judgments were made for the assigned active hand (first, third) and two for the passive hand (second, fourth).

##### Feedback session

In order to see whether participants' judgment would profit from experience, in a second session the Experimental-group (E) was instructed to try to fit their assigned active hand into the opening with vision being provided. Feedback was automatically delivered in case of a successful fit through: participants touched a to hand-length distance adjusted back-board, that triggered a bell. After experiencing one hand in the aperture (45+5 filler trials), subjects had to solve a judge-only block for the passive hand (36+4 filler trials). The Control-group had to solve all trials without the exposure, but with judgment only.

Please note, as described earlier for half of the total group the button pressing active hand was the dominant right hand and for the other half the assigned active hand was the non-dominant left hand. As described above we assigned an active hand because we wanted to test a paradigm suitable for unilateral stroke patients with hemiparesis of their left or right arm. We further divided the group according to condition into the experimental group that received feedback and the control group that did not receive feedback. In the experimental group only the active hand received feedback (again because patients would not be able to use their paretic arm). For half of the experimental group the active hand was the non-dominant hand. Thus, half of the experimental group received feedback for their dominant right hand and the other half of the experimental group experienced their non-dominant left hand in the aperture.

##### Size-estimation task

In the size-estimation task we assessed the ability for horizontal size perception. Subjects had to decide when a gradually adjusted opening width had the same size as the widest part of the hand (say stop with the allowance to correct). Horizontal start-openings were varied: In half of the 8 trials the horizontal width was gradually decreased starting from a 20 cm opening, in the other half openings were gradually increased starting from 0 cm. The vertical width was kept constant during the experimental conditions (set to individual's hand height). Left and right hand were presented in a fixed randomized order.

Half of the participants started with the control task first, half started with the affordance based judgments.

#### Data-analyses

The data-analysis was divided into two sections: section A. assessed the influence of different variables on overall judgment accuracy and response times (RT), and section B. used a detection theory approach.

##### ANOVA

*Judgment accuracy (%) and RT (ms)*. First we ran a control analysis to test potential confounding effects of group assignment and gender. An ANOVA with the variables group (Experimental/Control) and gender (male/female) was applied for the affordance based judgment task accomplished in the first session.

The greatest interest was in analyzing effects of *feedback* and *opening*. However, which hand had to be judged may additionally influence RT and judgment accuracy and potentially it also may affect how quickly feedback is integrated. Judgment accuracy for example could be modulated by whether the hand to be judged had to press the button or remained passive or whether in our right hand dominant sample the hand to be judged was left or right hand. We therefore ran two separate analyses taking these two variables of hand into account. *Hand dominance, opening* and/or *feedback* were fed into a repeated measures ANOVA with between subjects variable group (Experimental and Control) and within subjects variables hand (left/right), opening (−1.6, −0.8, −0.4, −0.2, ±0, +0.2, +0.4, +0.8, and +1.6 cm) and session (1 and 2). Further, a repeated measurements ANOVA was computed with between subjects variable group (Experimental and Control) and within subjects variables hand (active/passive), opening (−1.6, −0.8, −0.4, −0.2, ±0, +0.2, +0.4, +0.8, and +1.6 cm) and session (1 and 2).

*Analyzing size perception (cm)*. To see whether hand dominance, start-opening and/or gender played a role a repeated measures ANOVA was computed with between subjects variable gender (male/female) and within subject variables hand (left/right) and start-opening (0/20).

The correlation between size-perception and accuracy was analyzed (Pearson).

##### Detection theory approach

To analyze response tendencies we calculated subjects' discriminability, response bias and diagnostic accuracy with the help of detection theory (Green and Swets, 1966; Fox, 2004; Macmillan and Creelman, 2004).

*The discriminability index d*′. The discriminability index is a measure of the subjects' perceptual sensitivity that is independent of the criterion. The more sensitive the participant is at discriminating between reachable and non-reachable targets, the larger the d′ value will be. Its calculation is described below:

$$\begin{array}{l} {\text{d}}^{\prime }=\text{Z}(\text{Hit Rate})-\text{Z}(\text{False Alarm Rate}).\\ \text{False Alarm Rate}=(\text{No}.\text{ of False Alarms})\text{/}(\text{No}.\text{ of Actual}\\ \text{ Negative Cases)}\\ \text{Hit Rate}=(\text{No}.\text{ of Hits})\text{/}(\text{No}.\text{ of Actual Positive Cases}) \end{array}$$

Please note, to correct for Hit and False Alarm (FA) Rates of 0 (z would become infinite), we used the following standard correction (Macmillan and Creelman, 2004, p. 8):

$$\begin{array}{l} \text{If Hit Rate}=1:\text{Hit Rate}=1-1/(2^{*}\text{No}.\text{ of Actual Positive}\\ \text{ Cases}).\\ \text{If FA Rate}=0:\text{FA Rate}=1/(2^{*}\text{No}.\text{ of Actual Negative Cases}).\\ \text{If FA Rate}=1:\text{FA Rate}=1-1/(2^{*}\text{No}.\text{ of Actual Negative}\\ \text{ Cases}).\\ \text{If Hit Rate}=0:\text{Hit Rate}=1/(2^{*}\text{No}.\text{ of ActualPositive Cases}). \end{array}$$

*Response bias*. The participant's strategy is revealed by the sign of the response bias c. When c is negative the participant is liberal (i.e., responds Yes more often than the ideal observer). When c is positive the participant is conservative (i.e., responds No more often than the ideal observer). Its calculation is as follows:

$$\text{c}=-0.5^{*}[\text{Z}(\text{Hit Rate})+\text{Z}(\text{FA Rate})].$$

*ROC curves*. Diagnostic accuracy can be demonstrated by Receiver Operating Characteristic (ROC) curves. ROC curves represent a graphic description of how the Hit Rate of an observer changes as a function of changes in the False Alarm (FA) Rate. The Area Under the Curve (AUC) reflects perceptual accuracy by combining sensitivity and specificity into a single value. Plots representing perfect discrimination pass through the co-ordinates 0 and 1. These indicate 100% Sensitivity (Hit Rate, sensitivity) and Specificity (FA Rate, 1-specificity) and represent an AUC value of 1. According to an arbitrary guideline (based on a suggestion by Swets, 1988), one may distinguish between non-informative (<0.5), less accurate (0.5–0.7), moderately accurate (0.7–0.9), highly accurate (0.9) and perfect ratings (1).

### Results and discussion study1

#### Affordance based judgments

First we tested for confounding effects of group assignment (Experimental/Control) and gender (male/female) on affordance based judgments in the first session. This ruled out effects of both factors in the baseline accuracy- and RT-data [*F*_(1, 23)_ < 2.75, *p* > 0.11].

In order to evaluate the paradigm, the major goal was to test effects of group, opening, and session. Furthermore, to see whether hand dominance plays a role or whether there is an effect of hand indicating vs. not indicating the response two separate repeated measurements ANOVAs were computed with between subjects variable group (Experimental vs. Control) and within subjects variables hand [(left vs. right) or (active vs. passive)], opening (−1.6, −0.8, −0.4, −0.2, ±0, +0.2, +0.4, +0.8, and +1.6 cm) and session (1 vs. 2). For a detailed overview, please see Supplements (Supplementary Table 1 for descriptive statistics and Supplementary Table 2 for *F*- and *p*-values).

**Figure 2:**
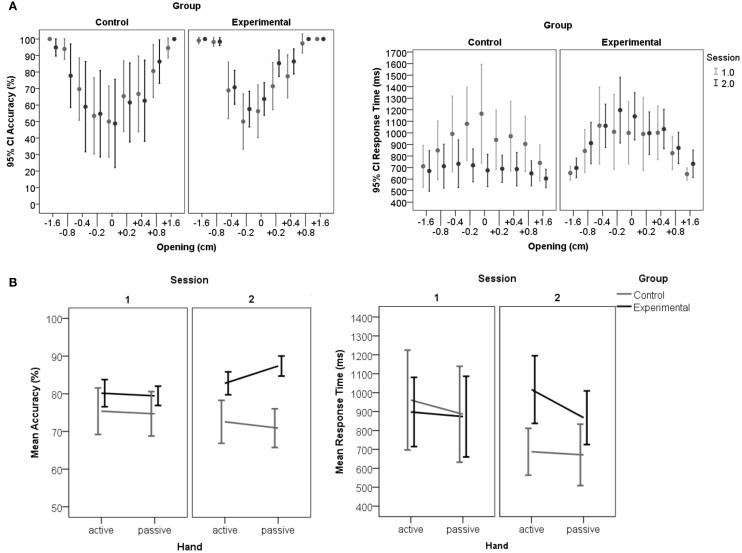
**Accuracy and RT in the aperture task. (A)** Displays accuracy (left) and RT (right) distributions across openings per group and session. The graphs show the main effect of opening with judgment accuracy being lower (left) and judgments being slower (right) for those openings that are close to the physical constraints of the hands' fit into the aperture (0 cm) compared to more extreme deviations (e.g. ±1.6 cm). The Experimental-group significantly increased judgment performance in session 2, which is most obvious for openings close to the hands'constraints. For trials ranging close to the constraints (openings: −0.2 to +0.4) the Control-group initiated responses faster in the second session than participants who received feedback (Experimental-group). Error-bars represent 95% confidence intervals. **(B)** Displays accuracy and response times per session, group and hand. Participants indicated responses with one designated hand (active hand), the other hand is called the passive hand. Left: In the second session the Experimental-group showed overall increased accuracy compared to the Control-group. Within the Experimental-group responses are significantly more accurate for the passive hand, which was judged subsequent to the learning experience with the active hand. The accuracy data implies that the improved criterion was transferred to the following passive hand trials. Right: Slower RTs for judgments when experiencing the active hand in the opening may reflect a reevaluation taking place during the feedback trials. Generally, faster responses in the second session within the Control-group suggest that this group based decisions on a judgment criterion settled in the first session. Error-bars represent 95% confidence intervals.

As expected, openings closer to the actual hand-size were judged more poorly and slower compared to more extreme openings (Figure 2A). The tests of within subjects contrasts show this main effect of opening to be quadratic [accuracy: *F*_(1, 25)_ = 461.20, *p* < 0.001; RT: *F*_(1, 25)_ = 30.62, *p* < 0.001].

The predicted judgment improvement after feedback is confirmed. The interaction session^*^group demonstrated an increase in accuracy in the second compared to the first session only for the Experimental-group [Bf-p = 0.025; *t*_(13)_ = −2.83, *p* = 0.014], but not for the Control-group [*t*_(12)_ = 1.66, *p* = 0.126; see Figure 2]. Further, it was found that in the second session the Control-group initiated faster responses compared to the Experimental-group [*t*_(24.8)_ = −2.87, *p* = 0.008]. The interaction with opening revealed that the RT advantage in the Control-group is specific to trials ranging close to the actual hand-fit for which decisions according to the accuracy results appear to be more difficult [opening^*^session^*^group interaction: Bf-p = 0.006; −0.2: *t*_(19.0)_ = 3.24, *p* = 0.004; 0: *t*_(22.6)_ = 4.09, *p* < 0.001; +0.2: 0: *t*_(21.8)_ = 3.06, *p* = 0.006; +0.4: *t*_(24.6)_ = 3.35, *p* = 0.003]. A possible explanation for these group-specific differences in the second session is the formation of a stable criterion during the first session. Only the Control-group was able to use a stabilized criterion in the second session, which may have enabled faster RT compared to the first session [*t*_(12)_ = 3.48, *p* = 0.005]. In contrast the Experimental-group had to reset the individual's criterion to integrate the feedback information and develop a new response strategy. This group therefore shows a similar response initiation in both sessions [*t*_(13)_ = −0.68, *p* = 0.509]. Importantly, no differences between groups were found for the first baseline session in RT or accuracy [Bf-p = 0.025; RT: *t*_(22.7)_ = 0.25, *p* = 0.806; accuracy: *t*_(17.7)_ = −1.71, *p* = 0.111].

The affordance based judgment task is thought to be solved by a higher order motor cognition capacity. We therefore were interested to see transfer effects induced from the hand experiencing feedback (active hand) toward the hand that was not exposed to the actual constraints of the aperture (passive hand). When distinguishing between active and passive hand an interaction between hand, session and group occurred for affordance judgment accuracy (Figure 2B). *Post-hoc* analyses demonstrated that within the Experimental-group responses in the second session are more accurate for the passive compared to the active hand [Bf-p = 0.004; *t*_(13)_ = −4.49, *p* = 0.001]. These results are at first sight puzzling, but can be explained by the way the experimental session is set up. Only the active hand is engaged in feedback trials. When judging for the active hand the participant is in the learning phase, whereas judgments for the passive hand are made in a subsequent block. Greater accuracy for the passive hand suggests that learning has been transferred.

In line with this, there is a tendency of the Experimental-group to respond slower with the active hand during the feedback trials compared to the following passive hand trials [Bf-p = 0.004; *t*_(13)_ = −2.82, *p* = 0.015]. The RT-delay for the active hand experiencing feedback in the Experimental-group can be explained by enhanced computational demands. After indication of the response, the participant is required to gently try to fit the hand into the opening in order to obtain feedback. RT may have been influenced by planning the subsequent action of the active hand toward the opening and participants had to update their evaluations according to feedback.

**Figure 3:**
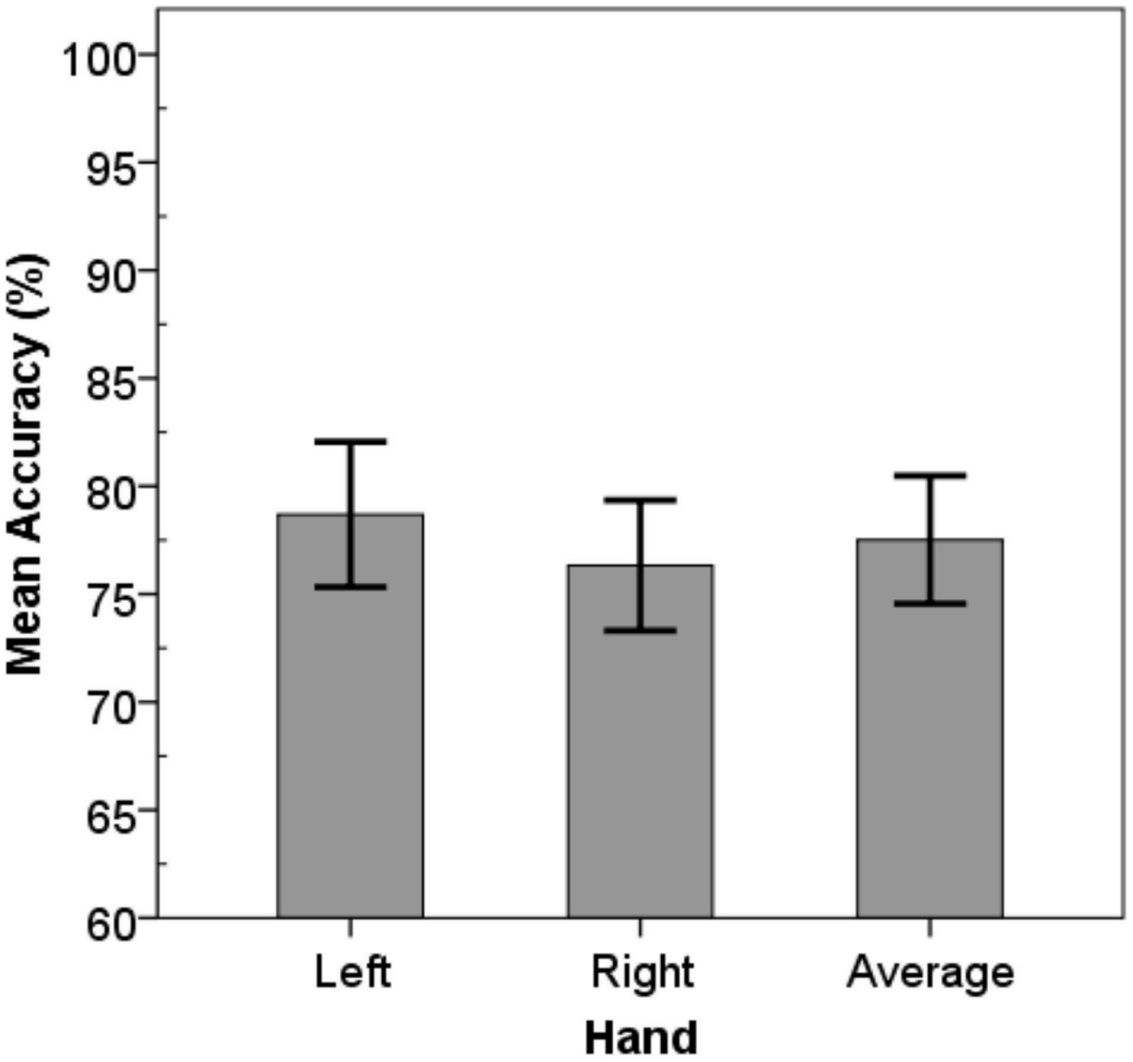
**Mean accuracy values**. Left hand judgments were significantly more accurate compared to right hand judgments. Error-bars represent 95% confidence intervals.

Against expectations there was no main effect of hand-dominance with respect to RT, instead an effect of hand dominance was found for accuracy (Figure 3). Surprisingly, judgments for the non-dominant left (*M* = 79.4%, *SD* = 8.3) compared to right hands (*M* = 76.8%, *SD* = 7.3) were significantly more accurate [*F*_(1.0, 25.0)_ = 5.83, *p* = 0.023].

Although, the interaction of hand^*^opening did not reach significance, there was a trend for subjects to respond “no” more often for the right compared to the left hand when it actually could fit through (increments: +0.2, + 0.4 cm). One may argue that judgments that involve actions with the right hand may be based on a larger hand representation. Notably, as will be shown hereafter, left and right hand did not differ significantly in size, and size-estimations for the two hands did not deviate. A conceivable explanation is that these judgment-errors for the right hand are not based on errors in size-estimation but based on the perception of extended action boundaries for the dominant hand. This has been suggested before based on evidence that subjects perceive themselves being able to grasp bigger objects with their right compared to their left hand (Linkenauger et al., 2009, 2011).

#### Size estimations

The average hand size was 9.7 cm (*SD* = 0.9), for male participants this was 9.9 cm (*SD* = 0.9), females' hands were 9.5 cm on average (*SD* = 0.8). Left (*M* = 9.6, *SD* = 0.9) and right (*M* = 9.7, *SD* = 0.9) hand sizes did vary within some participants but on a group level these did not differ significantly [*t*_(26)_ = 1.40, *p* = 0.174].

Size-estimations were defined by the difference of the estimated minus actual hand-size in centimeter (cm). On average, the size of the hands was overestimated (*M* = 1.05, *SD* = 1.0; Min = −0.61, Max = 3.35).

An ANOVA with gender (male/female), hand (left/right), and start-opening (0/20) was run. There was no significant effect of gender [*F*_(1, 25)_ = 0.30, *p* = 0.590]. Size-estimations for left and right hands did not differ [*F*_(1, 25)_ = 0.02, *p* = 0.879].

**Figure 4:**
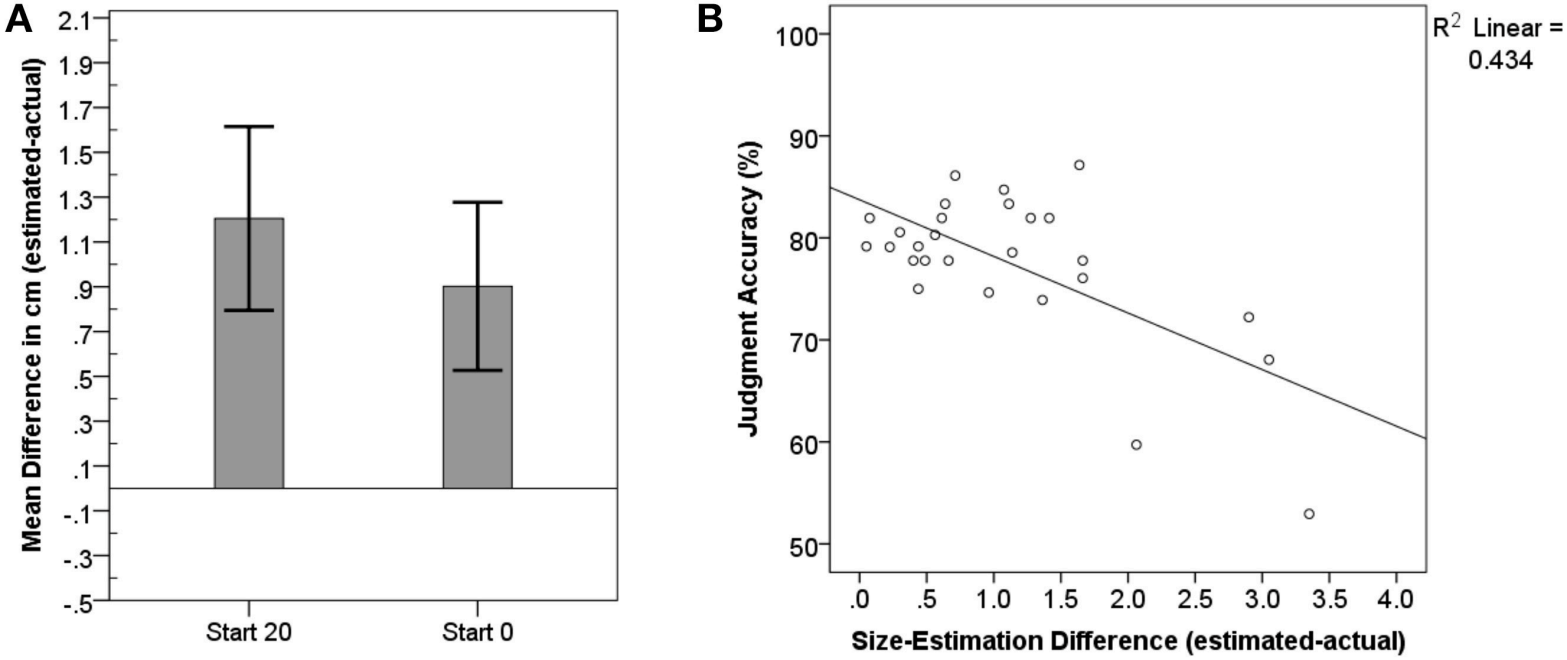
**Mean differences between estimated and actual hand size. (A)** Demonstrates that participants estimated their hands to be larger than they are, even more so when the aperture was gradually closed compared to when it was opened. Estimations for left and right hands did not differ. Error-bars represent 95% confidence intervals. **(B)** shows the significant correlation between correct judgment of whether the hand can fit into a presented opening and higher precision when estimating the hand size.

Despite that subjects were encouraged to adjust their first attempt whenever they felt it to be necessary, their estimations differed between the closing vs. opening adjustment. Hand-size-estimation for gradual outward-adjustments starting from 0 cm (*M* = 0.9, *SD* = 0.9) were significantly better than estimations for inward-adjustments starting from 20 cm [*M* = 1.2, *SD* = 1.0, *F*_(1, 25)_ = 13.85, *p* = 0.001; see Figure 4A]. This could potentially be attributed to firstly subjects overestimating their hand-size and secondly to their demand for safety. Participants may imagine their hands in the opening and respond with stop relatively fast for the closing aperture to ensure their hand's safety.

#### Correlations

We calculated correlations between actual average hand-size, size-estimations, accuracy-judgments, and RT for the aperture paradigm. For the size-estimation we first multiplied all negative differences with −1. The actual hand-size itself did not correlate significantly with accuracy (*r* = 0.27, *p* = 0.170) nor size-estimation (*r* = −0.34, *p* = 0.085).

None of the variables correlated significantly with RT (*p* > 0.523).

We found a significant correlation between the mean difference for size-estimations and accuracy in affordance judgments (Pearson: *r* = −0.66, *p* < 0.001). The more deficient the size-estimation the lower the affordance judgment accuracy (see Figure 4B). Interestingly, in accordance with size estimations being off by about 1 cm on average (*SD* = 1), the entire sample appears to achieve 100% accuracy between 0.8 and 1.6 cm for affordance based judgments.

#### Detection theory approach

**Figure 5:**
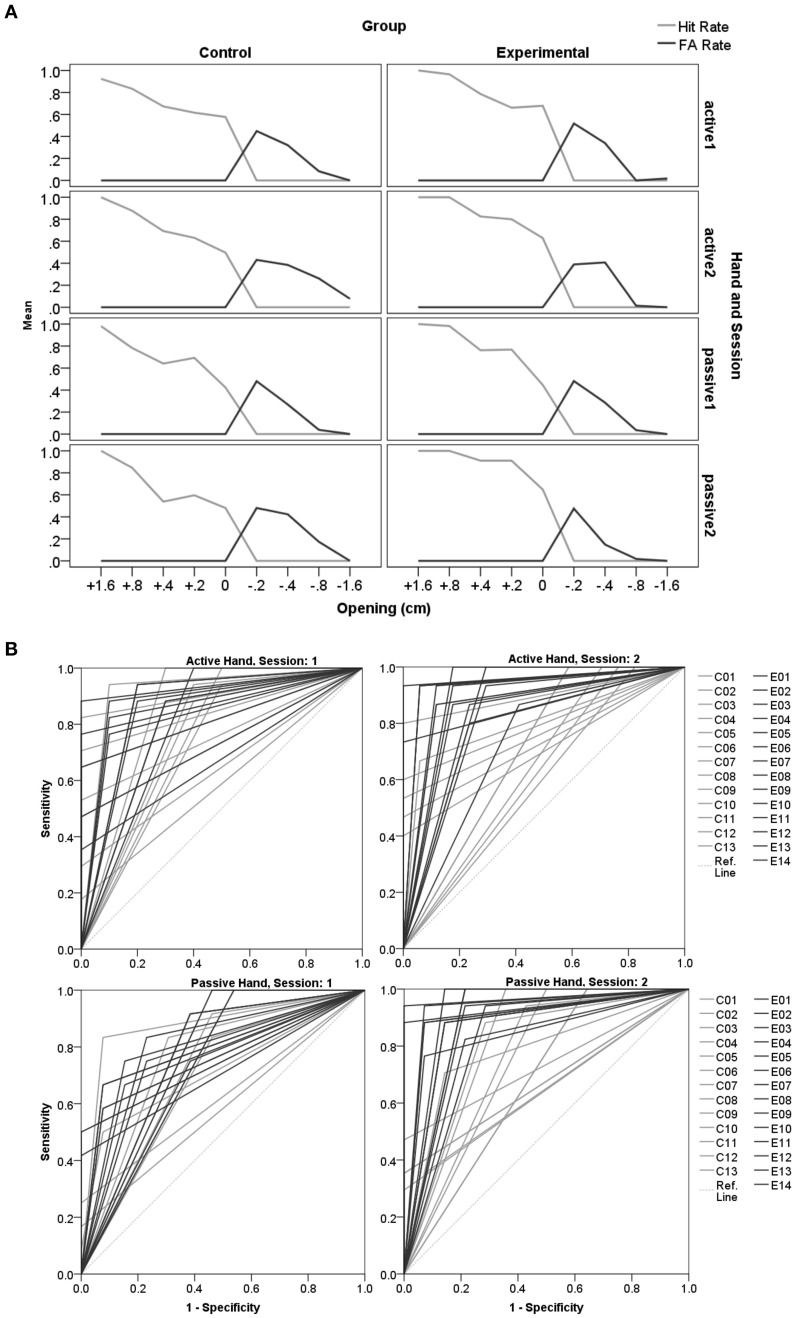
**Detection theory approach for the aperture task. (A)** Displays an overview of changes in Hit- and False Alarm Rates for the different openings across sessions (1, 2) for the active and the passive hand respectively. In **(B)** the ROC curves for individual participants are displayed (Control-group C: gray lines; Experimental-group E: black lines) for each session. 1-Specificity reflects the False Alarm Rate, and the Sensitivity indicates the Hit Rate. The reference line is at chance level (Ref. Line: light gray dots). The area under the curve is a measure of accuracy, which is perfect when FA = 0 and Hit = 1 (in the upper left corner). Accuracy significantly improved after feedback in session 2 for the subjects that were assigned to the Experimental compared to the Control-group.

Our main interest was in analyzing effects of feedback. The detection analysis confirms improvement in judgments for the passive hand as a result of preceding feedback experienced with the active hand in the Experimental-group only. The effect is shown through pairwise comparisons between sessions per group (Bf-p = 0.025). In the first session the mean criterion is close to zero (*M* = 0.05, *SD* = 0.68, *MD* = 0.15) indicating no extreme bias. However, there is a tendency of participants to erroneously say “no,” judging that their hand cannot fit into the aperture when it actually could. After feedback, a significant increase in Sensitivity [d′: *t*_(13)_ = −3.95, *p* = 0.002] and accuracy [AUC: *t*_(13)_ = −8.36, *p* < 0.001] is achieved for the passive hand in the Experimental-group. A major contribution to this improvement is the rise in the Hit Rate (equals a reduction of miss rate), which however, on its own does fail to reach statistical significance (Figure 5). Please see Supplementary Table 3 for further descriptive statistics and *t*- and *p*-values.

Some of our results are not in line with previous reports analyzing affordance based judgments when fitting one's own hand into an aperture (Ishak et al., 2008). We replicated that participants scaled their motor decisions to their body dimensions. However, while we find a close to ideal criterion with a tendency toward a rather conservative approach, Ishak et al. found that participants wedged their hands into apertures that were one centimeter smaller than their actual fit. Further, Ishak et al. did not find an effect of hand dominance, but in our study judgments for the right hand were significantly worse, reflecting a more conservative approach than with the left hand. The differential findings can be explained by differences in the approach. In the study by Ishak et al. affordance perception was measured in the rate of attempts to fit the hand through a diamond formed aperture. The authors point out that their experimental situation included low penalty for errors. Instead, participants were rewarded with candies when successfully reaching through the aperture to grasp the incentive.

## Study 2: reachability task

### Method

#### Participants

Nineteen individuals (12 female; mean age = 23 ± 2.6 years) from the University of Missouri participated in the two-session study. All participants were right-handed according to the Edinburgh Handedness Inventory (Oldfield, 1971), had normal or aid corrected-to-normal vision (at least 30 ft/9 m), and were naïve to the specific goals of the study. Participants provided informed consent in accordance with the local IRB and the Declaration of Helsinki. Participants solved two sessions. The study took approximately 2 h to complete (45–60 min per session), and participants received financial or study credit compensation. Next to the experimental tasks (approximately 30 min), participants completed the consent-form, a handedness questionnaire, a vision test and received study-debriefing. We randomly assigned half (*N* = 10) of the participants to an Experimental-group receiving feedback in the second session, the other half served as Control-group.

#### Materials and procedures

##### Material

**Figure 6:**
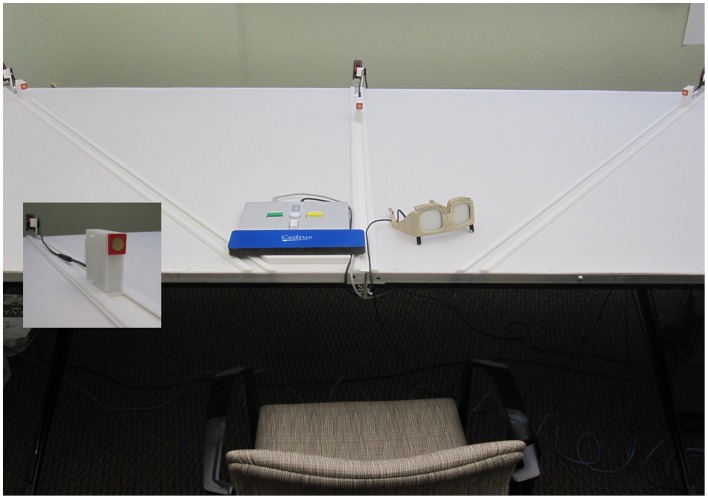
**Setting for judging reachability**. Participants were seated centrally in front of the height adjustable reaching apparatus, 25 cm from the table edge. The table's height was set underneath breast-level. Distance-measurements were possible using measurement-tapes mounted at the end of each track. Dimension specifications allowed for measuring millimeter increments. Participants wore plato-goggles (here: opaque) throughout the experiment to control for visual feedback and to allow for RT measurements. Goggles were opened at the beginning of each trial. The positioning of the response box reflects an affordance judgment block for the left side.

The custom made reaching apparatus consisted of a height adjustable table with three tracks mounted onto it as well as three rectangular objects with sensors (Figure 6). The objects could be manually moved within the tracks. On each track one object was presented. Object-distances were manipulated manually with the help of mounted measurement-tapes. The table height was adjusted to each participant's solar plexus. Participants were seated 25 cm away from the table on a rigid chair wearing a seatbelt. Participants wore plato-goggles throughout the experiment to control for visual feedback and to allow for RT measurements.

##### Measurements

Each session started out with measuring the maximum reach of one assigned side (left, right). Without vision participants had to push each object with their index-finger along the track as far as possible while bending forward was allowed but the bottom needed to stay seated. This was repeated 3 times and the maximum value was used for further settings. The seatbelt and table-edge prevented participants from falling, in case they would lose their equilibrium while reaching forward. For the measuring procedures the goggles were turned opaque to avoid visual feedback.

##### Affordance based judgments

In the affordance perception task participants had to judge whether a presented object was within reach. Reachability judgments were made for one assigned side of the body, the same side the subject pressed the response buttons with. The distance of the objects was varied using fixed negative and positive increments according to the maximum reachability (distance: −16, −8, −4, −2, ±0, +2, +4, +8, and +16 cm). Per session participants solved 54 (2 blocks × 3 tracks × 9 distances) plus 6 filler trials, resulting in a total of 60 trials. The 0-trials reflected the measurement, i.e., the individual's maximum reachability. To avoid an imbalance toward more frequent Yes-trials, we added filler trials for which the correct answer would be “No” (further than +16 cm).

In anticipation of applying this paradigm in stroke patients with potential hemiparesis, who are not able to use one arm, the task was solved for one side only. Participants in the current study indicated the response with the same side that they were to judge, e.g., if reaching ability was judged for the right arm, button presses were executed with the right hand and vice versa. Half of the group always indicated their responses via button press with their left hand, the other half with their right hand. The response box was positioned between two tracks, either left or right from the center depending on what side the participant had to judge for and press the buttons with. In anticipation of applying this paradigm in stroke patients with potential neglect, before the plato-goggles opened it was verbally communicated on which track the next object would be presented (left, right, and middle). This allowed orientation toward the correct side. The experiment started with 2 demonstration trials, presenting an extreme close or far distance on the left and right track.

In total subjects solved two sessions with each two blocks of 30 trials judging whether a given object was within reach (session1: block 1 and 2; session2: block 3 and 4).

##### Feedback session

In the second session all participants started out with an introductory block of 36 trials. In order to see whether participant's judgment would profit from experience, for this introductory block the Experimental-group (E) was instructed to first indicate their response as soon as the goggles opened and then try and reach toward each presented object. Feedback was automatically delivered in case of a successful touch of the sensor that was registered via the Superlab software and triggered a sound. The other half of the sample had to solve the same trials without the exposure, but with judgment only. After this introductory block (which was not included in data-analysis), all participants solved two more blocks with 30 trials each judging whether a presented object was within reach.

##### Depth-perception task

In the depth-perception task subjects had to decide when a gradually adjusted object on the track was aligned with a rigid object next to the track (say stop with the allowance to correct). Start-positions for the movable object on the track were set either about 8 cm before or behind the rigid object. Thus, for alignment, the object on the track was either gradually moved toward or away from the rigid object until the participant said stop. To cover the range of distances on each track the rigid object was presented for two distances: +16 and −16 cm from actual maximum reach. This results in a total of 12 trials (3 tracks × 2 start-positions × 2 distances). Trials were presented in a fixed randomized order.

Half of the participants started with the Depth-Perception task first, half started with the affordance judgments.

#### Data-analyses

Similar to study 1 the data-analysis is divided into two sections: section A. assesses the influence of different variables on overall judgment accuracy (%) and response times (RT), and B. using a detection theory approach. Below we describe the RT and accuracy variables assessed for the reachability paradigm.

##### ANOVA

*Judgment accuracy (%) and RT (ms)*. To test for confounding effects of group assignment and gender in the affordance perception task an ANOVA with the variables group (Experimental/Control) and gender (male/female) was run for the first session. Our main interest was to assess the effects of distance and feedback. In addition we analyzed potential effects of hand dominance and visual field (track). To allow for full cells two repeated measurements ANOVAs were computed. The first ANOVA included the between subjects variables hand (left/right) and group (Experimental and Control) and within subjects variables track (left/right/middle) and session (first vs. second). The second ANOVA included the between subjects variable group and within variables distance (−16, −8, −4, −2, ±0, +2, +4,+8, and +16 cm) and session (1, 2).

*Depth perception (cm)*. To see whether track, start-distance, and/or gender plays a role a repeated measurements ANOVA was computed with between subjects variable gender (male/female) and within subject variables track (left/right/middle) and start-position (+8/−8). Furthermore, the correlation between depth-perception and accuracy was analyzed (Pearson).

##### Detection theory approach

The used detection theory approach is identical to study1.

### Results and discussion study2

To test effects of group assignment and gender for the affordance perception task in the first session an ANOVA with the variables group (Experimental/Control) and gender (male/female) was run for accuracy- and RT-data, that ruled out effects of both factors [*F*_(1, 15)_ < 1.55, *p* > 0.23].

We ran two repeated measures ANOVAs to test effects of the variables group, track, hand, distance, and feedback on RT and accuracy. Please see Supplementary Table 4 for a full list of *F*- and *p*-values. We proposed that distances close to the actual reach are hardest to judge and that feedback will improve behavior.

**Figure 7:**
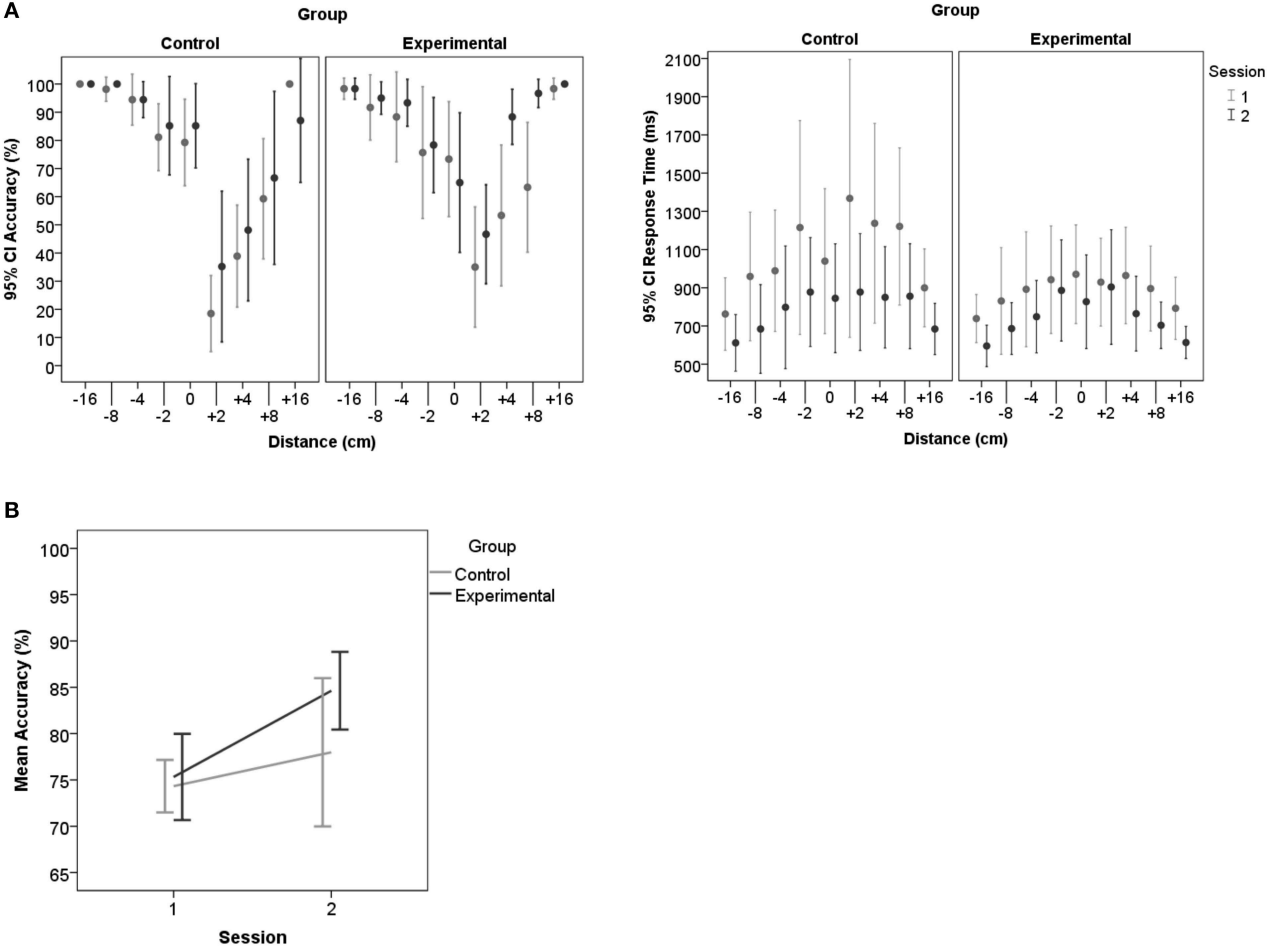
**Accuracy and RT per session and group for reachability judgments. (A)** Shows that participants overestimated how far they can reach by the skewed distribution of the accuracy measures. Accuracy for distances close to the maximum reach (0) decreased, especially for those that are out of reach (+2, +4 cm). **(B)** Demonstrates the judgment improvement in the second session, especially in the Experimental-group. Error-bars represent 95% confidence intervals.

We found a main effect of session [*F*_(1.0, 17.0)_ = 27.01, *p* = 0.006] and a quadratic effect of distance [*F*_(1.5, 25.3)_ = 27.01, *p* < 0.001] for accuracy as well as an interaction between the two variables [*F*_(2.5, 42.1)_ = 3.32, *p* = 0.037]. The group^*^session interaction did not reach significance. For RT, there was a main effect of session [*F*_(1.0, 17.0)_ = 11.88, *p* = 0.003] and distance [*F*_(2.6, 44.9)_ = 11.08, *p* < 0.001]. Paired testing for all distances or the interaction would result in too many comparisons, therefore we here refer to the descriptive images (Figure 7). As predicted accuracy appeared lowest and RTs delayed for distances that were slightly further away than actual maximum reachability. Moreover, reachability judgments were most accurate and quickest for distances close to the participant, replicating previous findings (Gabbard et al., 2007). The main effect of session demonstrates a general improvement in both groups. Participants improved and judged faster over the course of testing. In the first session judgments (accuracy: *M* = 74.8, *SD* = 5.2; RT: *M* = 961.4, *SD* = 382.0) were less accurate and slower compared to the second session (accuracy: *M* = 81.5, *SD* = 8.8; RT: *M* = 761.4, *SD* = 259.9). General improvement could be explained by repeated exposure to the task. Further, it may be possible that being exposed to the first session led to more conscious reaching in everyday life activities, which in turn may have led to uncontrolled feedback effects. These possibilities underline the necessity of including a control-group. However, the general trend toward improvement in both groups, as shown by the main effect, may have masked the role of feedback in the second session. To clarify this point we *post-hoc* compared the sessions within groups. In accordance with our hypothesis, only within the Experimental-group accuracy improved significantly in the second session, and despite the trend toward the same direction significance was not reached within the Control-group (Table 1).

**Table 1:** **Mean accuracy values (%) for each group compared *post-hoc* between sessions**.

**Group**	**Sessions**	**Mean**	**SD**
Control [*t*_(8)_ = −1.06, *p* = 0.320]	1	74.3	3.7
	2	78.0	10.4
Experimental [*t*_(9)_ = −3.93, *p* = 0.003]	1	75.3	6.5
	2	84.6	5.9

The second ANOVA included between subjects variables *hand* (left/right) and *group* (Experimental and Control) and within subjects variables *track* (left/right/middle) and *session* (first vs. second). The main effect of session is described above. Between subjects there was no effect of left vs. right hand assignments, therefore hand dominance does not seem to make a significant difference for reachability judgments. There was no effect of track for accuracy values. Although, participants were cued about where the object was presented before the goggles opened, RTs were significantly faster for objects presented on the middle track (*M* = 801.7, *SD* = 270.8) compared to objects presented on the left (*M* = 871.7, *SD* = 285.2) or right tracks [*M* = 899.4, *SD* = 332.6, *t*_(18)_ > 4.37, *p* < 0.001]. Except for the directional cues there was no particular instruction for how to position the head or eyes. Hence, this delay of responses toward the side could be due to shifting or calibrating the field of view into an optimal position for judging the laterally presented objects and/or due to an attentional shift from midline (Posner et al., 1980; Remington, 1980).

#### Depth perception

In the depth perception task subjects had to say stop as soon as the object moving along the track was aligned with an object positioned next to the track. The dependent variable was measured as the difference between the two objects resulting from the subject's verbally indicated adjustment. On average participants were able to make depth-perception adjustments with 1-mm accuracy (*SD* = 0.1, Min = −0.1, Max = 0.4).

**Figure 8:**
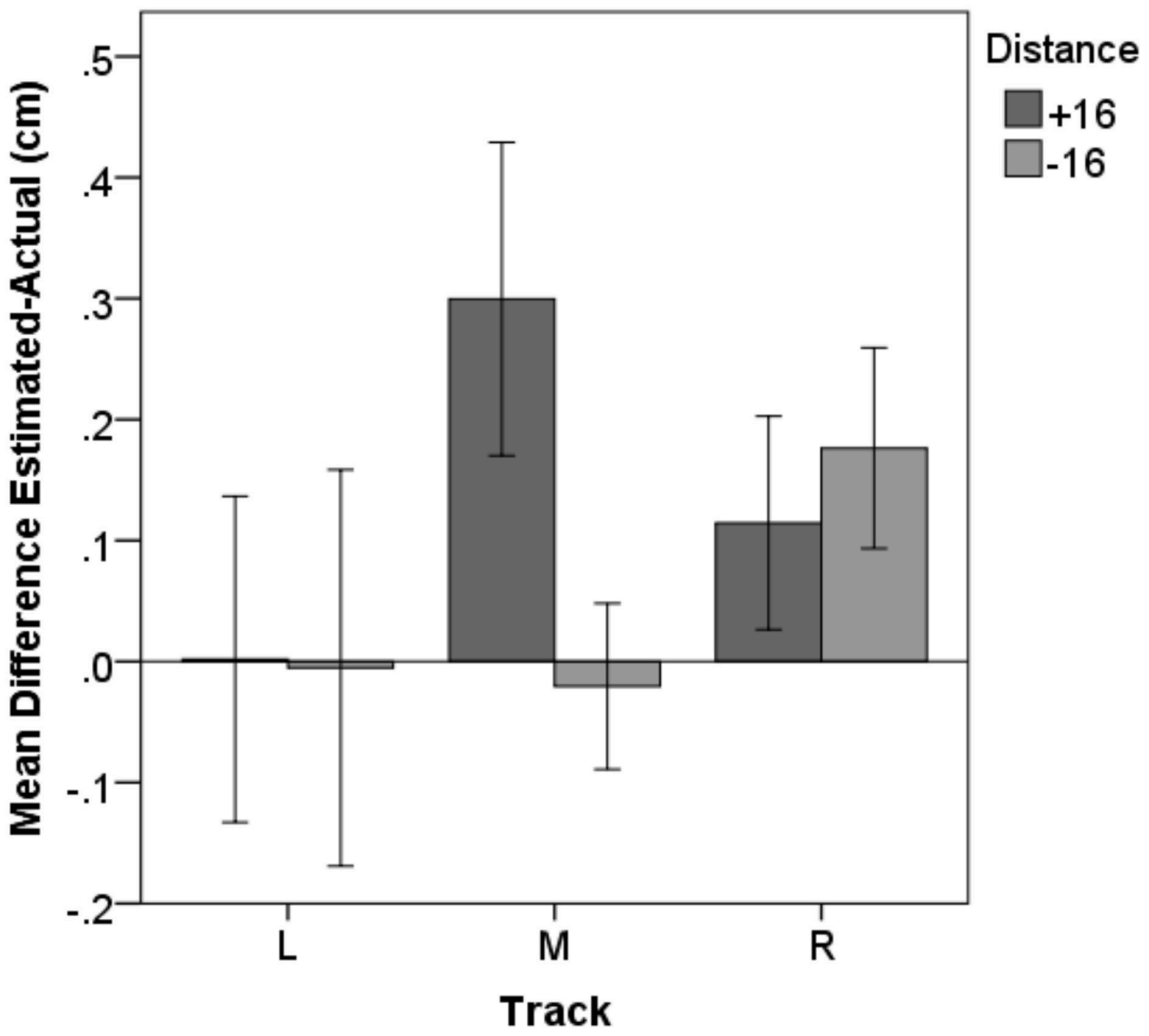
**Depth perception**. Subjects were able to judge whether two objects were aligned with less than 0.5 cm misestimating. For the middle track an effect of distance occurred, with participants' estimation being significantly worse for objects presented further away. Error-bars represent 95% confidence intervals.

A repeated measures ANOVA was run with within subjects factors track (left, right, and middle) and presented distance of the fixed object (+16 cm or −16 cm from the participant's maximum reach). The analysis revealed an interaction between distance and track [*F*_(1.9, 33.6)_ = 10.90, *p* < 0.001]. Pairwise comparison demonstrated that only for the middle track judgments were significantly worse for objects presented far-away (*M* = 0.29 cm, *SD* = 0.27) compared to those nearby [*M* = −0.02 cm, *SD* = 0.14, *Bf* = 0.005, *t*_(18)_ = 4.44, *p* < 0.001; see Figure 8]. The miss-estimation for the far-away objects is consistent with an underestimation of depth (Saunders and Backus, 2006).

#### Correlations

We calculated correlations between actual average maximum reach, depth-estimations, arm-length estimation and accuracy-judgments, and RT for the reach paradigm. For the estimation-values we first multiplied all negative differences with −1. Participants' arm-length was measured from the shoulder to the index finger-tip. The maximum reach has been measured from the table-end and therefore lower values reflect further reaches and cause the correlation with arm-length to be negative. At the end of the study participants were asked to estimate their arm-length. Most subjects overestimated their arm-length with a mean error of 4.8 cm, but it needs to be pointed out that participants' responses were very variable (*SD* = 7.7, Min = −7.8, Max = 22.0 cm).

The correlations confirm that the longer the participants' arms are the further they can actually reach (*r* = −0.715, *p* = 0.001). Against our expectation none of these perceptual measures correlated with accuracy or RT for reachability judgments,—this includes depth perception.

#### Detection theory approach

**Figure 9:**
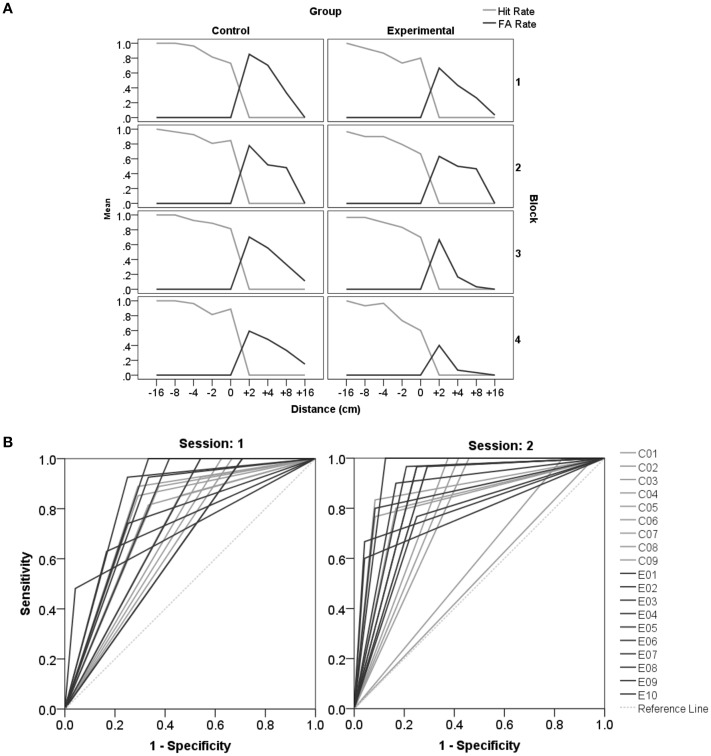
**Detection theory approach for reachability judgments. (A)** Displays an overview of changes in Hit- and FA Rates for the different distances across all blocks (session1: block1 and 2, session2: block 3 and 4). **(B)** Displays the ROC curves for individual participants (C, Control-group; E, Experimental-group) for each session. Accuracy improves significantly after feedback in the second session.

Importantly, the detection analysis confirmed improvements in affordance based judgments due to feedback as demonstrated by pairwise comparisons between sessions per group (Bf-p = 0.025). In the Control-group none of the variables of the detection theory approach demonstrated differences between sessions. In the Experimental-group the FA Rate significantly dropped in the second session [*t*_(9)_ = 2.75, *p* = 0.022], as can best be seen in Figure 9A. Hit Rates did not differ between sessions. Accordingly, in session 2 the sensitivity value d′ [*t*_(9)_ = −3.83, *p* = 0.004] and the AUC [*t*_(9)_ = −3.56, *p* = 0.006; Figure 9B] increased significantly for the Experimental-group demonstrating an improvement in accuracy after feedback. In line with these results the Experimental-group revealed a trend of the criterion changing over time from a liberal (*M* = −0.42, *sd* = 0.67) toward an ideal strategy (*M* = −0.09, *sd* = 0.44). In the Control-group the mean criterion remained at the same liberal level in session 1 (*M* = −0.62, *sd* = 0.35) and 2 (*M* = −0.61, *sd* = 0.70). Please see Supplementary Table 5 for further descriptive statistics and *t*- and *p*-values.

## General discussion

Affordance perception encompasses determining action opportunities for a given setting. It comprises a dynamic integrating process of cognitive components involved in perception and action necessary to gauge and update the relationship between relevant body constraints and environmental properties. Thus, affordance perception resembles a multifaceted construct, probably engaging a complex neural network of components involved in motor cognition. Stroke may affect appropriate affordance based judgments on different levels, due to changed body constraints or impaired cognitive functions. Our goal was to test an affordance based judgment paradigm in healthy young adults with the perspective to be applied in stroke patients. We used simple instructions, a limited number of trials (doable within 30 min), and took into account difficulties with attention to the contralateral hemi-space as well as the use of only one hand. To specify setting dependent estimations we studied two tasks. With two independent samples of young healthy adults, we investigated the ability to judge the fit of one's hand in an aperture (study1) and the ability to judge whether objects are within reach (study2) based on the same approach.

Overall, we confirmed that settings close to the actual measure were judged more poorly and slower compared to more extreme settings, replicating prior results (Gabbard et al., 2007). In accordance with prior studies participants overestimated their reaching capacities (Carello et al., 1989; Mark et al., 1997; Gabbard et al., 2007). However, when participants judged whether their hand can fit into an aperture the current results demonstrated a rather conservative approach.

In order to study potential accuracy-improvements in affordance based judgments, in a second session we analyzed effects of task exposure and feedback. In line with our predictions and findings of previous affordance perception related studies (Mark and Vogele, 1987; Mark et al., 1990; Weast et al., 2011), we here demonstrate that subjects' accuracy improved. This was most obvious for more error-prone settings close to the bodily constraints.

Furthermore, according to the affordance theory, perception of environmental properties (such as size, or depth perception) is a fundamental component. Therefore, we also analyzed correlations of these abilities with the capability to explicitly judge action opportunities. As predicted size-estimations correlated with the accuracy in determining whether the hand can fit into an aperture. However, performance in the depth perception task did not correlate with reachability judgments.

**Affordance perception strongly depends on the setting and task at hand**.

Due to the between subjects design it needs to be noted that comparisons between the two affordance based judgment tasks need to be interpreted with some caution. However, we strived to use similar measurement approaches for both the reaching and the aperture task.

The present data suggests that the mechanisms involved in affordance perception strongly depends on the task. In study2 participants overestimated their reachability while being seated in accordance with prior reachability studies (Carello et al., 1989; Mark et al., 1997; Gabbard et al., 2007). In contrast study1 shows rather conservative response tendencies when subjects decided whether their hand fits into an aperture,—despite the fact that we used a similar setting and measurement approach for either task. Note however, Ishak et al. (2008) rewarded their participants with candies when they were able to reach through diamond shaped apertures and found a very liberal response tendency. Thus, even when solving a similar task, the response criterion may vary depending on the setting, including the risks and benefits that participants may attribute as a consequence to their behavior.

**The ability to perceive relevant perceptual properties only partly explains performance in affordance based judgments. Its role for affordance perception may depend on the degrees of freedom in a task**.

The ability to perceive and estimate hand-size was quite accurate in our sample. For judging whether a hand can fit into an aperture, size perception seems to be a strong determinant. Study2 demonstrated that participants were very good at perceiving depth, but very variable when estimating their arm-length. In contrast to our aperture study, these measurements of perceiving environmental and bodily properties did not correlate with affordance based judgment accuracy or RT. This is in line with other studies indicating that different processes are involved in visual depth perception and visually directed action tasks (e.g., Loomis et al., 1992). Still one can assume that preserved depth perception is a prerequisite for the here described affordance perception task. A possible explanation for the weak correlation between perceiving environmental properties and affordance based judgments could be the number and impact weight of single properties defining certain action opportunities. In contrast to the reachability task, affordance perception for the aperture task does not require taking as many properties into account. For example, when deciding upon the hand's fit, the movement component of bending forward to the target should not have much impact, instead the judgment is predominantly based on information about the size of the hand and the opening. More generally the hand may be used as a stable perceptual metric for scaling objects that afford actions,—an argument that recently has been similarly formulated by Linkenauger et al. (2014). In their study Linkenauger et al. magnified the size of different body parts and objects to the same degree and demonstrated that subjects perceived their hand as less magnified than other body parts or objects. However, hand size perception cannot explain the entire construct of affordance perception when judging whether a hand can fit into an aperture. Against expectations, the analysis reveals that affordance based judgments for the non-dominant left hand were significantly more accurate compared to judgments for the dominant right hand. Interestingly, although size-estimations correlated with accuracy-judgments, this left vs. right hand accuracy-judgment difference was not found for size-estimations, suggesting that only partly overlapping mechanisms are used to solve the two tasks.

**Participants integrate newly acquired knowledge**.

Further, differences between the two tasks occur in the second session. When deciding whether the hand can fit into an aperture the Control-group produced faster RTs during the second session compared to the first session and compared to the Experimental-group. This may indicate that in the second session the Control-group retrieved a represented evaluation strategy that was developed during the first session. Whereas feedback during session 2 requires the Experimental group to integrate newly acquired knowledge.

In contrast to the aperture task, in study2 general exposure to the task seemed to improve accuracy in reachability judgments and led to quicker responses in both the Control and Experimental-groups. It is feasible that participants integrated knowledge acquired from exposure to daily life reaching between sessions, therefore leading to a trend toward improvement in the Control-group as well. Thus, the argument about the Control-group using a stabilized criterion can only be made for the aperture task. This is in line with the idea of the hand-size being commonly used as a stable criterion when judging action opportunities,—unless an update by direct experience is provided. New knowledge can be integrated into the representation as demonstrated by the performance increase of the Experimental-group in the second session. For the reachability task instead it seems that participants cannot revert to such a stable criterion. This may be explained by higher degrees of freedom in this task.

Despite of these differences between the two settings, signal detection analysis underlines an important similarity, namely that feedback plays a significant role in improving affordance based decisions. Feedback in the Experimental-group had a significantly advantageous effect on judgment accuracy, which is most obvious for more error-prone settings close to the bodily constraints, i.e., actual hand-fit or maximum reachability. In the aperture task, the Experimental-group judged best for the passive hand, following the learning trials with the active hand, demonstrating transfer toward the untrained hand.

**The results have implications for research in patients with brain damage**.

One major novelty of the study is provided by the method which was developed for future assessment of affordance perception in patients with brain damage. Thus, an important gain from this study are the paradigms themselves. As described in the introduction, stroke for example may endanger appropriate affordance perception on different levels,—by immensely changed bodily capabilities after damage to motor relevant brain areas, by impaired insight into the disorder as well as by problems with action planning or problems with perceiving object and spatial properties.

Aside from the proposed diagnostic value, we also demonstrated that the paradigm has the potential for training applications. In line with previous studies (e.g., Weast et al., 2011) we demonstrated that feedback can change perception of affordances. With two studies each testing a unique affordance perception task we show for the first time that training of affordance perception can improve detection measures independent of the prior trend of response tendencies. On the one hand our studies illustrated that feedback can lead to changes from a previously liberal to a rather ideal strategy when judging reachability in a seated position. Or in case of conservative tendencies for judging the fit of ones hand in an aperture, it can lead to an improvement in sensitivity measures. The results have important implications for neurorehabilitation of patients with unilateral brain damage and new bodily constraints. For one it is good news that updates and improvements of judgments seem to be possible even for healthy participants with quite accurate judgments. However, the transfer toward the untrained limbs as demonstrated in the aperture task needs to be regarded with caution. First, further studies need to test for generalization of this statement toward other tasks. Second, if the same abilities are attributed to both sides of the body, then learning with the intact hand may be problematic for patients with asymmetrical motor functions. Patients with residual motor functions for example may have to be trained regularly with both hands in these types of tasks in order to adequately adapt affordance based judgments to the asymmetrical motor functions while also taking into account possible motor-rehabilitation progress.

While we successfully tested our paradigm in healthy young adults it needs to be noted that a subsequent patient study will require an age matched healthy control-group. For one, body capabilities change while growing older, and secondly cognitive skills may decline. Aging appears to affect affordance perception, as demonstrated by several studies investigating reachability (Gabbard et al., 2011; Gabbard and Cordova, 2013). Alarmingly, falls are reported to correlate with reduced reaching capabilities in elderly adults (Butler et al., 2011).

To our knowledge, this project presents the first approach to assess affordance perception accounting for the challenges of working with a stroke patient population. We introduce two tasks that clearly have daily life relevance. Subpopulations with particular difficulties may be identified and it could be tested whether controlled feedback leads to a reduction of erroneous judgments. We conclude that future applications of the paradigm should include a patient population and a healthy age matched control-group to diagnose potential difficulties with affordance perception after brain injury, as well as the effects of training.

## Funding

This work was funded by the James S McDonnell Foundation Collaborative Activities Award (grant no. 220020190) to SHF and a EU FP7 Marie Curie Zukunftskolleg Incoming Fellowship Programme, University of Konstanz (grant no. 291784) awarded to JR.

### Conflict of interest statement

The authors declare that the research was conducted in the absence of any commercial or financial relationships that could be construed as a potential conflict of interest.

## Footnotes

^1^While all increments here are treated as if fitting exactly into yes/no-response–categories it needs to be noted that actual capability can sometimes vary minimally across repeated trials. This applies to those settings that are one increment lower or higher than the actual capability measure.
