# Supplementary material for: Diagnostics and Training of Affordance Perception in Healthy Young Adults—Implications for Post-Stroke Neurorehabilitation
Source: Front Hum Neurosci. 2016 Jan 6;9:674. doi: 10.3389/fnhum.2015.00674 (PMC4701931; doi:10.3389/fnhum.2015.00674)
Supplement: Supplementary file 1 [file Table1.DOCX]

Supplementary table 1. Aperture-paradigm: Descriptive statistics for accuracy and RT. The table includes descriptive values split for group (Control and Experimental), hand [(active and passive)/(left and right)] and session (1 and 2).

|  |  |  | **Accuracy** | | **Response Times** | |
| --- | --- | --- | --- | --- | --- | --- |
| **Group** | **hand** | **session** | **Mean** | **SD** | **Mean** | **SD** |
| Control (N=13) | active | 1 | 75.4 | 10.2 | 960.7 | 436.9 |
|  |  | 2 | 72.6 | 9.4 | 687.4 | 205.1 |
|  | passive | 1 | 74.7 | 9.8 | 885.8 | 419.8 |
|  |  | 2 | 70.9 | 8.5 | 670.9 | 268.8 |
|  | left | 1 | 75.7 | 10.3 | 939.7 | 453.4 |
|  |  | 2 | 73.1 | 9.3 | 641.7 | 197.8 |
|  | right | 1 | 74.4 | 9.6 | 906.7 | 404.9 |
|  |  | 2 | 70.3 | 8.5 | 716.7 | 268.8 |
| Experimental (N=14) | active | 1 | 80.2 | 6.2 | 897.7 | 317.1 |
|  |  | 2 | 82.8 | 5.3 | 1016.2 | 309.5 |
|  | passive | 1 | 79.5 | 4.4 | 873.3 | 369.5 |
|  |  | 2 | 87.4 | 4.6 | 867.3 | 245.7 |
|  | left | 1 | 81.5 | 5.4 | 845.3 | 310.9 |
|  |  | 2 | 86.8 | 5.1 | 931.4 | 306.7 |
|  | right | 1 | 78.1 | 4.9 | 925.7 | 370.5 |
|  |  | 2 | 83.3 | 5.3 | 952.1 | 271.7 |
