# Supplementary material for: Diagnostics and Training of Affordance Perception in Healthy Young Adults—Implications for Post-Stroke Neurorehabilitation
Source: Front Hum Neurosci. 2016 Jan 6;9:674. doi: 10.3389/fnhum.2015.00674 (PMC4701931; doi:10.3389/fnhum.2015.00674)
Supplement: Supplementary file 2 [file Table2.DOCX]

Supplementary table2. Aperture-paradigm: Outcomes of the repeated measures ANOVA for accuracy and response times. The table displays statistics for between subjects variable group (Experimental and Control) and within subjects variables opening (-1.6, -0.8, -0.4, -0.2, +/-0, +0.2, +0.4, +0.8, +1.6 cm), session (1 and 2) and hand. The table includes results of both RM ANOVAS with variables of either hand dominance (l/r hand) or active vs. passive hand (a/p hand).

| **Variable** | **Factor** | **df** | **F** | **p** |
| --- | --- | --- | --- | --- |
| **Accuracy** | opening | 1.2, 30.1 | 16.44 | .000** |
|  | session | 1.0, 25.0 | 0.40 | .531 |
|  | opening * session | 2.0, 49.6 | 0.93 | .400 |
|  | group | 1.0, 25.0 | 17.522 | .000** |
|  | opening * Group | 1.2, 1.0 | 0.59 | .480 |
|  | session * Group | 1.0, 1.0 | 10.32 | .004** |
|  | opening * session * Group | 2.0, 1.0 | 0.84 | .435 |
|  | l/r hand | 1.0, 25.0 | 5.83 | .023* |
|  | l/r hand * opening | 4.0, 99.6 | 2.34 | .060 |
|  | l/r hand * session | 1.0, 25.0 | 0.38 | .545 |
|  | l/r hand * opening * session | 3.3, 82.7 | 1.57 | .199 |
|  | l/r hand * Group | 1.0, 1.0 | 0.25 | .619 |
|  | l/r hand * opening * Group | 4.0, 1.0 | 0.49 | .742 |
|  | l/r hand * session * Group | 1.0, 1.0 | 0.12 | .733 |
|  | l/r hand * opening * session * Group | 3.3, 1.0 | 1.16 | .332 |
|  | a/p hand | 1.0, 25.0 | 0.11 | .738 |
|  | a/p hand * opening | 3.5, 88.3 | 3.02 | .027* |
|  | a/p hand * session | 1.0, 25.0 | 2.10 | .160 |
|  | a/p hand * opening * session | 3.3, 81.9 | 1.65 | .179 |
|  | a/p hand * Group | 1.0, 1.0 | 1.08 | .309 |
|  | a/p hand * opening * Group | 3.5, 1.0 | 1.46 | .225 |
|  | a/p hand * session * Group | 1.0, 1.0 | 4.70 | .040* |
|  | a/p hand * opening * session * Group | 3.3, 1.0 | 1.31 | .274 |
| **RT** | opening | 2.6, 64.7 | 17.71 | .000** |
|  | session | 1.0, 25.0 | 2.50 | .126 |
|  | opening * session | 2.9, 71.9 | 1.85 | .148 |
|  | group | 1.0, 25.0 | 1.09 | .308 |
|  | opening * Group | 2.6, 1.0 | 1.95 | .138 |
|  | session * Group | 1.0, 1.0 | 6.43 | .018* |
|  | opening * session * Group | 2.9, 1.0 | 3.32 | .026* |
|  | l/r hand | 1.0, 25.0 | 1.93 | .177 |
|  | l/r hand * opening | 4.1, 101.4 | 0.97 | .429 |
|  | l/r hand * session | 1.0, 25.0 | 0.49 | .490 |
|  | l/r hand * opening * session | 3.6, 90.9 | 0.77 | .539 |
|  | l/r hand * Group | 1.0, 1.0 | 0.38 | .541 |
|  | l/r hand * opening * Group | 4.1, 1.0 | 2.27 | .066 |
|  | l/r hand * session * Group | 1.0, 1.0 | 3.72 | .065 |
|  | l/r hand * opening * session * Group | 3.6, 1.0 | 0.98 | .416 |
|  | a/p hand | 1.0, 25.0 | 7.58 | .011* |
|  | a/p hand * opening | 4.0, 99.4 | 0.80 | .529 |
|  | a/p hand * session | 1.0, 25.0 | 0.39 | .536 |
|  | a/p hand * opening * session | 3.7, 91.3 | 1.39 | .247 |
|  | a/p hand * Group | 1.0, 1.0 | 0.49 | .490 |
|  | a/p hand * opening * Group | 4.0, 1.0 | 1.65 | .168 |
|  | a/p hand * session * Group | 1.0, 1.0 | 4.30 | .048* |
|  | a/p hand * opening * session * Group | 3.7, 1.0 | 0.60 | .646 |
