# Supplementary material for: Diagnostics and Training of Affordance Perception in Healthy Young Adults—Implications for Post-Stroke Neurorehabilitation
Source: Front Hum Neurosci. 2016 Jan 6;9:674. doi: 10.3389/fnhum.2015.00674 (PMC4701931; doi:10.3389/fnhum.2015.00674)
Supplement: Supplementary file 3 [file Table3.DOCX]

Supplementary table 3. Aperture-paradigm: Detection theory approach. The table displays descriptive statistics and pairwise comparisons between sessions for each group.

| Group | Variable | Hand | session | Mean | SD | t | df | p |
| --- | --- | --- | --- | --- | --- | --- | --- | --- |
| Control | FA Rate | Active | 1 | 0.23 | 0.23 | -1.55 | 12 | .147 |
|  |  | Active | 2 | 0.30 | 0.35 |  |  |  |
|  |  | Passive | 1 | 0.20 | 0.17 | -1.43 | 12 | .178 |
|  |  | Passive | 2 | 0.28 | 0.26 |  |  |  |
|  | Hit Rate | Active | 1 | 0.72 | 0.27 | -0.45 | 12 | .663 |
|  |  | Active | 2 | 0.74 | 0.26 |  |  |  |
|  |  | Passive | 1 | 0.69 | 0.28 | 0.17 | 12 | .868 |
|  |  | Passive | 2 | 0.68 | 0.31 |  |  |  |
|  | c | Active | 1 | 0.12 | 0.88 | 0.92 | 12 | .374 |
|  |  | Active | 2 | -0.01 | 1.08 |  |  |  |
|  |  | Passive | 1 | 0.16 | 0.83 | 0.70 | 12 | .498 |
|  |  | Passive | 2 | 0.03 | 1.01 |  |  |  |
|  | d' | Active | 1 | 1.82 | 0.56 | 0.26 | 12 | .802 |
|  |  | Active | 2 | 1.79 | 0.56 |  |  |  |
|  |  | Passive | 1 | 1.72 | 0.51 | 1.09 | 12 | .299 |
|  |  | Passive | 2 | 1.59 | 0.32 |  |  |  |
|  | AUC | Active | 1 | 0.78 | 0.10 | 0.83 | 12 | .423 |
|  |  | Active | 2 | 0.75 | 0.11 |  |  |  |
|  |  | Passive | 1 | 0.73 | 0.07 | -0.04 | 12 | .966 |
|  |  | Passive | 2 | 0.73 | 0.07 |  |  |  |
| Experimental | FA Rate | Active | 1 | 0.22 | 0.14 | 0.43 | 13 | .673 |
|  |  | Active | 2 | 0.20 | 0.11 |  |  |  |
|  |  | Passive | 1 | 0.21 | 0.17 | 0.83 | 13 | .420 |
|  |  | Passive | 2 | 0.16 | 0.09 |  |  |  |
|  | Hit Rate | Active | 1 | 0.82 | 0.17 | 0.71 | 13 | .489 |
|  |  | Active | 2 | 0.85 | 0.09 |  |  |  |
|  |  | Passive | 1 | 0.79 | 0.15 | -2.15 | 13 | .051 |
|  |  | Passive | 2 | 0.89 | 0.07 |  |  |  |
|  | c | Active | 1 | -0.09 | 0.56 | 0.10 | 13 | .925 |
|  |  | Active | 2 | -010 | 0.36 |  |  |  |
|  |  | Passive | 1 | 0.02 | 0.61 | 0.78 | 13 | .449 |
|  |  | Passive | 2 | -0.14 | 0.35 |  |  |  |
|  | d' | Active | 1 | 1.92 | 0.32 | -1.12 | 13 | .282 |
|  |  | Active | 2 | 2.03 | 0.41 |  |  |  |
|  |  | Passive | 1 | 1.91 | 0.21 | -3.95 | 13 | .002* |
|  |  | Passive | 2 | 2.41 | 0.46 |  |  |  |
|  | AUC | Active | 1 | 0.83 | 0.07 | -1.48 | 13 | .162 |
|  |  | Active | 2 | 0.87 | 0.07 |  |  |  |
|  |  | Passive | 1 | 0.76 | 0.03 | -8.36 | 13 | .000* |
|  |  | Passive | 2 | 0.89 | 0.05 |  |  |  |
