# Supplementary material for: Diagnostics and Training of Affordance Perception in Healthy Young Adults—Implications for Post-Stroke Neurorehabilitation
Source: Front Hum Neurosci. 2016 Jan 6;9:674. doi: 10.3389/fnhum.2015.00674 (PMC4701931; doi:10.3389/fnhum.2015.00674)
Supplement: Supplementary file 4 [file Table4.docx]

Supplementary table 4. Reachability-paradigm: ANOVA results for both accuracy (%) and response time (ms). The table displays ANOVA results of the between subjects variables hand (left/right) and group (Experimental and Control) and within subjects variables track (left/right/middle) and session and the ANOVA results of between subjects variable group and within variables distance (-16, -8, -4, -2, +/-0, +2, +4,+8, +16 cm) and session (1 and 2).

| **Variable** | **Factor** | **df** | **F** | **p** |
| --- | --- | --- | --- | --- |
| **Accuracy** | session | 1.0, 15.0 | 8.98 | .009 |
|  | track | 1.6, 24.0 | 0.10 | .861 |
|  | session * track | 1.7, 25.2 | 0.61 | .524 |
|  | group | 1.0, 15.0 | 2.61 | .127 |
|  | group * session | 1.0, 1.0 | 1.58 | .228 |
|  | group * track | 1.6, 1.0 | 0.01 | .973 |
|  | group * session * track | 1.7, 1.0 | 0.56 | .547 |
|  | l/r hand | 1.0, 15.0 | 1.94 | .184 |
|  | l/r hand * session | 1.0, 1.0 | 0.12 | .730 |
|  | l/r hand * track | 1.6, 1.0 | 0.84 | .421 |
|  | l/r hand * session * track | 1.7, 1.0 | 0.06 | .918 |
|  | group * l/r hand * track | 1.6, 1.0 | 2.27 | .134 |
|  | group * l/r hand * session | 1.0, 1.0 | 0.03 | .873 |
|  | group * l/r hand * session * track | 1.7, 1.0 | 1.65 | .215 |
|  | session | 1.0, 17.0 | 9.95 | .006 |
|  | distance | 1.5, 25.3 | 27.01 | .000 |
|  | session * distance | 2.5, 42.1 | 3.32 | .037 |
|  | group | 1.0, 17.0 | 2.29 | .149 |
|  | group * session | 1.0, 1.0 | 1.99 | .176 |
|  | group * distance | 1.5, 1.0 | 2.50 | .114 |
|  | group * session * distance | 2.5, 1.0 | 1.59 | .212 |
| **RT** | session | 1.0, 15.0 | 12.00 | .003 |
|  | track | 1.7, 25.0 | 14.80 | .000 |
|  | session * track | 1.3, 1.0 | 0.82 | .405 |
|  | group | 1.0, 15.0 | 0.43 | .520 |
|  | group * session | 1.0, 1.0 | 1.54 | .233 |
|  | group * track | 1.7, 1.0 | 1.85 | .183 |
|  | group * session * track | 1.3, 1.0 | 0.56 | .504 |
|  | l/r hand | 1.0, 15.0 | 0.59 | .455 |
|  | l/r hand * session | 1.0, 1.0 | 0.69 | .418 |
|  | l/r hand * track | 1.7, 1.0 | 1.40 | .262 |
|  | l/r hand * session * track | 1.3, 1.0 | 1.02 | .346 |
|  | group * l/r hand * track | 1.7, 1.0 | 1.04 | .357 |
|  | group * l/r hand * session | 1.0, 1.0 | 0.00 | .979 |
|  | group * l/r hand * session * track | 1.3, 1.0 | 0.13 | .779 |
|  | session | 1.0, 17.0 | 11.88 | .003 |
|  | distance | 2.6, 44.9 | 11.08 | .000 |
|  | session * distance | 3.0, 50.3 | 0.84 | .477 |
|  | group | 1.0, 17.0 | 0.63 | .437 |
|  | group * session | 1.0, 1.0 | 1.54 | .232 |
|  | group * distance | 2.6, 1.0 | 1.25 | .302 |
|  | group * session * distance | 3.0, 1.0 | 1.67 | .186 |
