# Supplementary material for: Diagnostics and Training of Affordance Perception in Healthy Young Adults—Implications for Post-Stroke Neurorehabilitation
Source: Front Hum Neurosci. 2016 Jan 6;9:674. doi: 10.3389/fnhum.2015.00674 (PMC4701931; doi:10.3389/fnhum.2015.00674)
Supplement: Supplementary file 5 [file Table5.DOCX]

Supplementary table 5. Reachability-paradigm: Detection theory approach. The table displays descriptive statistics and pairwise comparisons between sessions for each group.

| Group | Variable | session | Mean | SD | t | df | p |
| --- | --- | --- | --- | --- | --- | --- | --- |
| Control | FA Rate | 1 | 0.46 | 0.14 | 0.64 | 8 | .539 |
|  |  | 2 | 0.41 | 0.30 |  |  |  |
|  | Hit Rate | 1 | 0.89 | 0.06 | -0.56 | 8 | .594 |
|  |  | 2 | 0.91 | 0.08 |  |  |  |
|  | c | 1 | -0.62 | 0.35 | -0.07 | 8 | .946 |
|  |  | 2 | -0.61 | 0.70 |  |  |  |
|  | d' | 1 | 1.5 | 0.2 | -1.18 | 8 | .273 |
|  |  | 2 | 1.8 | 0.7 |  |  |  |
|  | AUC | 1 | 0.73 | 0.05 | -0.86 | 8 | .416 |
|  |  | 2 | 0.76 | 0.12 |  |  |  |
| Experimental | FA Rate | 1 | 0.38 | 0.22 | 2.75 | 9 | .022* |
|  |  | 2 | 0.18 | 0.09 |  |  |  |
|  | Hit Rate | 1 | 0.84 | 0.18 | -0.20 | 9 | .849 |
|  |  | 2 | 0.85 | 0.13 |  |  |  |
|  | c | 1 | -0.42 | 0.67 | -1.75 | 9 | .114 |
|  |  | 2 | -0.09 | 0.44 |  |  |  |
|  | d' | 1 | 1.6 | 0.3 | -3.83 | 9 | .004* |
|  |  | 2 | 2.2 | 0.4 |  |  |  |
|  | AUC | 1 | 0.75 | 0.07 | -3.56 | 9 | .006* |
|  |  | 2 | 0.84 | 0.05 |  |  |  |
